# Hypomethylation of *IL6ST* promotes development of endometriosis by activating JAK2/STAT3 signaling pathway

**DOI:** 10.1371/journal.pone.0317569

**Published:** 2025-01-16

**Authors:** Yue Hu, Hailong Chen, Lijuan Jin, Xiumei Chi, Jian Zhao, Qinying Cao

**Affiliations:** 1 Hebei Medical University, Shijiazhuang, Hebei, People’s Republic of China; 2 Department of Gynecology, Shijiazhuang People’s Hospital, Shijiazhuang, Hebei, People’s Republic of China; Teikyo University, School of Medicine, JAPAN

## Abstract

Endometriosis is a chronic inflammatory disorder characterized by presence of endometrial tissue outside the uterine cavity. Immunohistochemical analysis (IHC) revealed markedly elevated expression of *IL6ST* in endometrial tissue of patients with ovarian endometriosis. Level of methylation of *IL6ST* is diminished in patients with endometriosis, whereas level of mRNA expression is markedly elevated by RT-PCR. Cell Counting Kit-8, Transwell, Terminal deoxynucleotidyl transferase dUTP nick end labeling assays substantiated endometrial stromal cells stably transfected with 3*FLAG-*IL6ST* plasmid exhibited enhanced viability, augmented invasive capacity, and notable reduction in apoptosis rates. Furthermore, *IL6ST* facilitated progression of endometriosis by activating mitogen-activated protein kinase 9/Signal Transducer and Activator of Transcription 3 signaling pathway. Western blot analysis revealed significantly elevated protein levels of p-JAK2/JAK2, p-STAT3/STAT3, HIF-1α, and VEGF in *IL6ST* overexpression group. Conversely, JAK2/STAT3 inhibitor WP1066 had markedly reduced p-JAK2 and p-STAT3 protein levels in *IL6ST* overexpression group. Inhibiting JAK2/STAT3 signaling pathway had mitigating effect on proliferative and invasive enhancement of endometrial stromal cells, as well as inhibition of apoptosis induced by *IL6ST*. These findings offer novel potential targets and strategies for the treatment of endometriosis.

## Introduction

Endometriosis (EM) is chronic inflammatory condition characterized by ectopic growth of endometrial cells outside uterus. Estrogen-dependent inflammatory process affects approximately 6–10% of women [1, 2]. Endometriosis is prevalent gynecological condition, characterized by persistent or cyclical pelvic pain; infertility resulting from pelvic adhesions, tubal obstruction, or ovarian dysfunction [3]; dysmenorrhea due to prolonged menstrual periods and increased menstrual flow [4]; chocolate cysts can precipitate pelvic discomfort, particularly during menstruation, and may give rise to complications such as adhesions and infertility [5]; and potential emotional states such as anxiety and depression. Current treatments mainly include pharmacological therapy [6], surgical intervention [7], and assisted reproductive technologies [8], though the precise causes of endometriosis remain unclear.

Progression of endometriosis is believed to be result of abnormal activation of endometrial stromal cells. Among these, DNA methylation is one of endometrial biology’s most prevalent epigenetic modifications. Numerous investigations have illustrated direct correlation between DNA methylation levels and the expression of genes linked to uterine disorders [9, 10]. Abnormal DNA methylation patterns have been identified in ectopic endometrial tissues, with over 40,000 CpG (Cytosine-phosphate-Guanine) dinucleotides showing differential methylation in genomes of ectopic endometrial cells compared to normal endometrial cells [11]. Specific DNA methylation/demethylation patterns in stromal cells are responsible for the suppression or overproduction of certain proteins. Epigenetic modifications in stromal cells contribute to increased expression and accumulation of inflammatory and tissue-remodeling substances [12]. Significant changes in DNA methylation observed in endometrial stromal cells are marked by increased CpG island density. DNA methyltransferases add methyl groups to CpG islands, inhibiting transcription factor binding and reducing promoter activity [13]. Potential methylation regions can be predicted using specialized software in the field of bioinformatics [14].

*IL6ST*, also known as GP130 (Glycoprotein 130), is signaling molecule essential for the cytokine signaling pathway. Although *IL6ST* does not directly bind cytokines, it transmits signals into cell through receptor complexes. In IL-6 signaling pathway, IL-6 binds to IL-6R (IL-6 receptor), and this complex subsequently associates with *IL6ST*, initiating downstream signaling [15]. Alterations in *IL6ST* methylation are believed to contribute to cardiovascular disease and promote systemic inflammation [16]. Variations in *IL6ST* methylation levels have been linked to enhanced downstream stearoyl-CoA activity, which is significantly associated with the development of adipose tissue inflammation [17]. During progression of colitis, IL6/GP130/STAT3(Signal Transducer and Activator of Transcription 3) signaling pathway is upregulated in colonic epithelium, promoting development of colitis. Upregulation of JAK2(Janus kinase 2)/signal transducer and activator of transcription 3 pathway has also been observed in endometriosis. Studies have shown that JAK inhibitor tofacitinib can mitigate exacerbation of endometriosis [18]. Administration of STAT inhibitors has been shown to alleviate severity of endometriosis in mouse models while suppressing IL-6 signaling pathway and diminishing transforming growth factor-beta (TGF-β) levels [19]. In analogous signaling pathway studies, activation of *IL6ST* and IL-6 genes was found to activate JAK2/STAT3 signaling pathway [20]. Persistent activation of STAT3 by *IL6ST* signaling has been associated with development of fibrosis in endometriosis [21].

Comparative analysis of clinical ovarian endometrial tissue with normal uterine tissue using RT-PCR revealed significant differential methylation in promoter region of IL6ST, indicating markedly increased *IL6ST* expression and heightened methylation status in endometriosis. High methylation at the 37th position in the promoter region constitutes hotspot for CpG islands. Despite numerous reports on epigenetic modifications in endometriosis, there is relatively limited research specifically addressing the methylation of *IL6ST* molecule. Given that changes in *IL6ST* methylation status influence progression of various diseases, coupled with our laboratory’s previous sequencing results, we aim to validate the role of *IL6ST* in endometriosis and its related pathways. However, the specific mechanisms remain elusive.

## Materials and methods

### Clinical samples

From 1/10/2020 to 31/8/2023, samples were collected from 46 samples of ectopic endometrium and endometrial tissue from 11 normal women. Final date of access for data was 31/8/2024. The cohort of 46 patients with ectopic endometrium ranged in age from 17 to 51 years, with an average age of 37.44±6.98 years. Those with normal endometrial tissue ranged from 31 to 47 years, with an average age of 37.27±4.91 years. These data were collected at the Shijiazhuang People’s Hospital. All patients exhibited regular menstrual cycle and had not received any hormone therapy for minimum of six months before surgery. All tissue samples (normal endometrium group, n = 11; endometriosis group, n = 46) were collected during surgery and promptly preserved in liquid nitrogen for subsequent analysis. The experimental segment involving patients in this study received approval from the Ethics Committee of Shijiazhuang People’s Hospital. The ethics approval number is Yanke Lunshen 2022 No. 025, and informed consent was obtained from all participants.

### Immunohistochemistry

Tissue samples underwent immunohistochemical staining for IL-6ST. Tissues were fixed in formalin, embedded in paraffin, and sectioned at thickness of 5 μm. Sections were deparaffinized in xylene, rehydrated through series of graded ethanol solutions, and incubated with 3% hydrogen peroxide blocking solution. Following blocking step, primary antibodies were incubated at 4°C overnight. Biotinylated secondary antibodies, specifically goat anti-rabbit IgG (RGAR001, Proteintech, Wuhan, China), were then added and incubated at 37°C for 40 min. Next, horseradish peroxidase-conjugated streptavidin solution was applied and incubated at 37°C for 40 min. Sections were developed using 3,3’-diaminobenzidine (DAB) solution, counterstained with hematoxylin for 3 to 10 min, blued in tap water for an additional 5 to 10 min, dehydrated through series of graded alcohols, cleared in xylene, and mounted with neutral gum. Bright-field observations were performed using inverted microscope. IHC staining for each sample was evaluated by two pathologists (J.N.C. and A.S.), who reached consensus. The mean optical density (IOD/AREA) was calculated using Image-Pro Plus 6.0 software (Media Cybernetics, Inc., Rockville, MD, USA).

### Cell culture and transfection

Primary human endometrial stromal cells (obtained from T0533, ABM) were cultured in DMEM/F12 medium containing L-glutamine, HEPES, and sodium pyruvate at 37°C in 5% CO₂ incubator. IL-6ST overexpression recombinant plasmid lentivirus was constructed using the GV657 plasmid, which was purchased from GeneChem (Shanghai, China). Endometrial stromal cells were transfected with the GV675-IL-6ST lentivirus to establish stable IL-6ST overexpressing cell lines (*IL6ST*-OE).

### Sodium bisulfite modification via polymerase chain reaction (PCR)

In total volume of 18 μL of water, sample DNA (50–1000 ng) was denatured by adding 2 μL of 3 M sodium hydroxide and incubating the mixture at 37°C. Subsequently, 278 μL of 4.8 M sodium bisulfite and 2 μL of 100 mM hydroxyquinoline were added to denatured sample, and mixture was incubated in thermal cycler for 15 min at 55°C and 30 sec at 95°C for 20 cycles. Modified DNA was then desalted using the QIAquick PCR purification protocol. Desulfonation was initiated by adding 5.5 μL of 3 M sodium hydroxide and 5 μg of glycogen to the mixture, which was incubated at 37°C for 15 min. DNA was precipitated with 5.6 μL of sodium acetate and 150 μL of 100% ethanol. The precipitate was washed with 70% ethanol and dissolved in 30–50 μL of TE buffer (10 mM Tris–HCl, pH 8, 1 mM EDTA). Reaction products were amplified using primers specific to the modified *IL6ST* DNA, as detailed in **Table 1**.

**Table 1:** The primer sequence for *IL6ST*.

Primers	Sequences
*IL6ST*-F	ACCCACAAGCCAGCCAACAG
*IL6ST*-R	AAACAAAGGAGCACATAGCCCAAAG
Left F primer (*IL6ST*)	TGAAGGTAGTAGTTGAAGTTATGT
Right R primer (*IL6ST*)	ATTCTCTACTAACTTTTATCTCCAAACTAT
Right S primer (*IL6ST*)	AAAGAAGTTGGTATTTAAAAAG

### RT-qPCR

Total RNA was extracted using Trizol-TriQuick Reagent RNA extraction kit (Solarbio, R1100, Beijing, China). cDNA was synthesized from 2 μg RNA using the FastKing One-Step Genomic DNA Removal and cDNA Synthesis Premix (Tiangen, KR118, Beijing, China) and stored at -80°C. Quantitative polymerase chain reaction (qPCR) was conducted using SuperReal PreMix Plus (Tiangen, FP205, Beijing, China) with glyceraldehyde-3-phosphate dehydrogenase (GAPDH) as internal control. The primer sequences are presented in **Table 1** for reference.

### CCK-8 assay

Influence of IL-6ST overexpression on the proliferation of endometrial stromal cells was assessed using CCK-8 kit (Solarbio, CA1210-500T, Beijing, China). Treated endometrial stromal cells were seeded in 96-well plates at a density of 2*10^3^ cells per well, with 200 μL medium, and incubated at 37°C. At 0, 24, 48, and 72 h after adherence, 110 μL of medium containing 10 μL CCK-8 solution was added to each well, followed by incubation at 37°C for 1.5 h. Absorbance was measured at 450 nm using microplate reader.

### Transwell assay

Matrigel (Solarbio, M8370, Beijing, China) was pre-coated, and 5×10⁴ cells from different treatment groups were resuspended in serum-free medium and seeded into the upper chamber of Transwell (354480, Corning, NY, USA). Lower chamber was filled with 700 μL of complete medium. Following 36 h incubation period at 37°C in 5% CO₂ incubator, the inserts were removed, and the cells on the upper surface were wiped off with cotton swabs. Cells were then stained with 0.1% crystal violet for 25–30 min, after which they were gently washed and counted under microscope.

### TUNEL assay

Detection of apoptosis in endometrial stromal cells was conducted using the One-Step TUNEL Apoptosis Assay Kit (C1090, Beyotime, Beijing, China). Following 24 h incubation period, cells were fixed in 4% paraformaldehyde for 30 min and permeabilized with 0.3% Triton X-100. TUNEL reaction mixture (50 μL) was added, and cells were incubated in the dark at 37°C for 1 h. Presence of apoptotic cells was then confirmed through observation under fluorescence microscope.

### Western blot

Cells were collected and lysed with WB lysis buffer at 4°C for 30 min, after which they were subjected to centrifugation at 12,000 rpm for 15 min. Protein concentration was determined using the Pierce™ BCA Protein Assay Kit (23227, Thermo Scientific™, Massachusetts, USA). Protein samples (20 μg) were subjected to SDS-PAGE and subsequently transferred to PVDF membranes (IPVH00010, Immobilon™-P, Darmstadt, Germany) at 60 V for 3 h. Membranes were blocked with 5% milk for one hour, incubated with primary antibodies at 4°C overnight, and then with secondary antibodies at room temperature for one hour. Following antibodies were used for detection: following antibodies were used: anti-IL-6ST (67766-1-Ig, Proteintech, Wuhan, China), anti-GAPDH (AB181602, Abcam, Cambridge, UK), Phospho-Jak2 (ab32101, Abcam, Cambridge, UK), anti-JAK2 (ab108596, Abcam, Cambridge, UK). The following antibodies were used: phospho-Stat3 (#9138, CST, Boston, Massachusetts, USA), anti-Stat3 (#4904, CST Boston, Massachusetts, USA), anti-HIF-1α (ab1, Abcam, Cambridge, UK), anti-VEGF (FNab09391, FineTest, Wuhan, China). GAPDH was employed as Internal Control. Intensity of the bands was quantified using ImageJ software.

### Data analysis

Data analysis was conducted using GraphPad PRISM 10.1. Differences between the two groups were compared using an independent sample t-test, while one-way ANOVA was used for comparisons among multiple groups. Immunohistochemical images were analyzed for integrated optical density (IOD) and pixel area (AREA) using Image-Pro Plus 6.0 software to determine mean density (IOD/AREA). Band intensities from Western blots were quantified using Image J software. Data were presented as mean ± standard deviation (SD). P-value < 0.05 was considered statistically significant.

## Results

### Elevated expression of *IL6ST* in endometrial tissue of ovarian EMs patients

**Fig 1:**
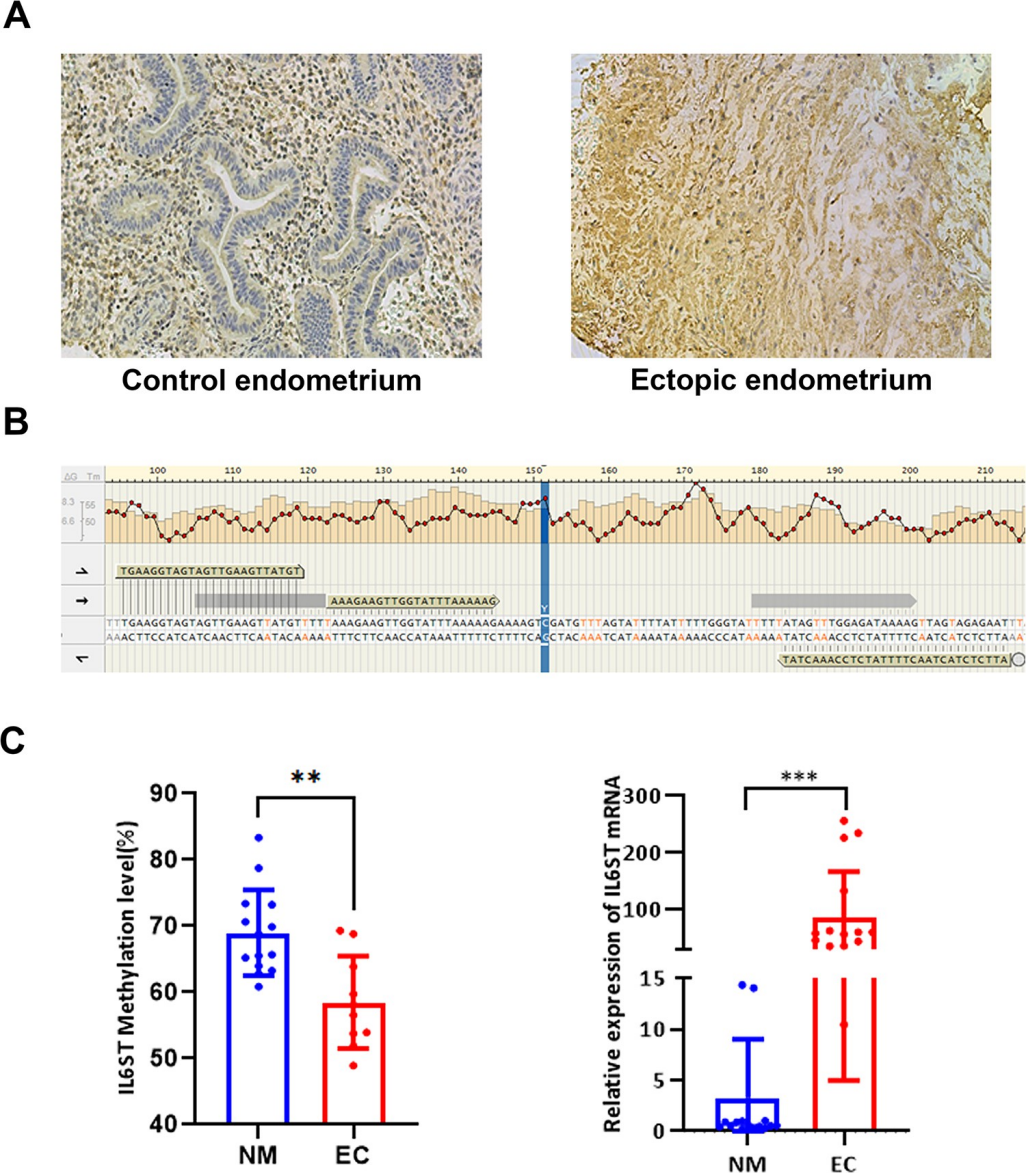
Immunohistochemical staining of *IL6ST* in endometrial tissues (SP×200). (A) Statistical analysis of the optical density values of *IL6ST* in immunohistochemical examinations between normal endometrium and EC patients. (B) The methylation sites of *IL6ST* were predicted within the region chr5:55286053–55286653, with a detailed map provided. The specific site of methylation is located at position 55286353. (C) Examination of the variations in *IL6ST* methylation levels and mRNA expression through PCR between normal endometrium and EM patients. Statistical analysis was performed using t-tests, *P<0.05, **P<0.01, ***P<0.001.

Using Illumina Human Methylation 450K BeadChip, we compared global methylation patterns of in situ and ectopic endometrium from six ovarian EM patients with six control endometrial samples. Analysis revealed 205 differentially methylated promoter regions between in situ endometrium and control endometrium, and 412 such regions between ectopic endometrium and control endometrium [22]. To evaluate protein expression of *IL6ST* in endometriosis and normal endometrial tissues, immunohistochemical staining results revealed significantly higher expression of *IL6ST* in ectopic endometrial tissues compared to normal endometrial tissues (NM) (Table 2). These findings indicate that *IL6ST* is prominently expressed in endometriosis, with notable preponderance expression in the cytoplasm compared to the nucleus and other cellular subregions. Moreover, it is plausible that *IL6ST* is activated prior to the commencement of aberrant endometrial growth and proliferation, with its expression intensifying during the malignant transformation of endometriosis (Fig 1A). Methylation sites of *IL6ST* were predicted from chr5:55286053–55286653 and prediction map of these sites was provided. Methylation site is located at position 55286353 (Fig 1B). Outcomes of the methylation PCR revealed notably diminished methylation degree of *IL6ST* in the ectopic endometrial cohort as contrasted with the normal endometrial cohort. Additionally, RT-PCR outcomes highlighted marked elevation in the mRNA content of *IL6ST* in the ectopic endometrial group relative to normal endometrial group (Fig 1C).

**Table 2:** IHC results of *IL6ST*.

Group	Mean Density (Mean ±SD)
(NM)	0.0027±0.00051
(EC)	0.0092±0.00191**

Note

** indicates significant difference compared to the negative control group.

### *IL6ST* overexpression promotes proliferation and invasion of endometrial stromal cells while inhibiting apoptosis

**Fig 2:**
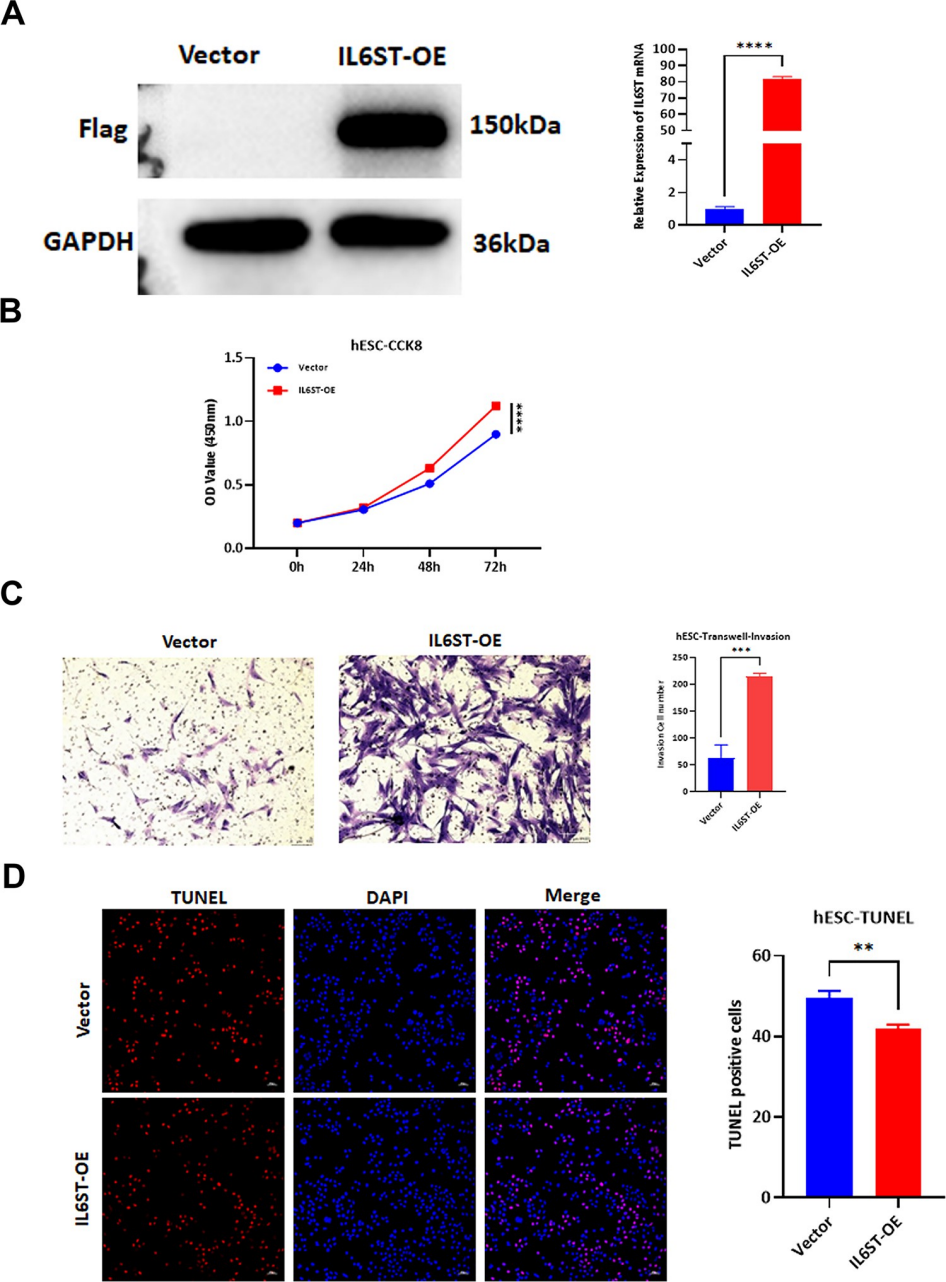
Effects of *IL6ST* overexpression on the proliferation, invasion, and apoptosis of endometrial stromal cells. (A) Western Blot and RT-PCR analysis of *IL6ST* protein and RNA levels. (B) CCK8 assay of cell proliferation. (C) Transwell assay of cell invasion. (D) TUNEL assay of cell apoptosis. Statistical analysis was performed using t-tests, *P<0.05, **P<0.01, ***P<0.001.

To elucidate role of *IL6ST* in pathogenesis of endometriosis, Western blot and RT-PCR analyses revealed protein and mRNA levels of 3*FLAG-*IL6ST* were significantly heightened in *IL6ST*-infected endometrial stromal cells (Fig 2A). CCK8 assays substantiated significant increment in proliferative potential of endometrial stromal cells post stable transfection with 3FLAG-*IL6ST*, as manifested by considerably higher absorbance at 450 nm after 72 hours cells transfected with the empty vector (Fig 2B). Transwell assays revealed fourfold augmentation in number of invasive cells in *IL6ST* overexpression (OE) group compared to cells transfected with empty vector group (Fig 2C). TUNEL assays indicated significant diminution in number of apoptotic cells in the *IL6ST*-OE group, achieving ratio of one-fifth that of empty vector group (Fig 2D).

### *IL6ST* overexpression activates the JAK2/STAT3 signaling pathway

**Fig 3:**
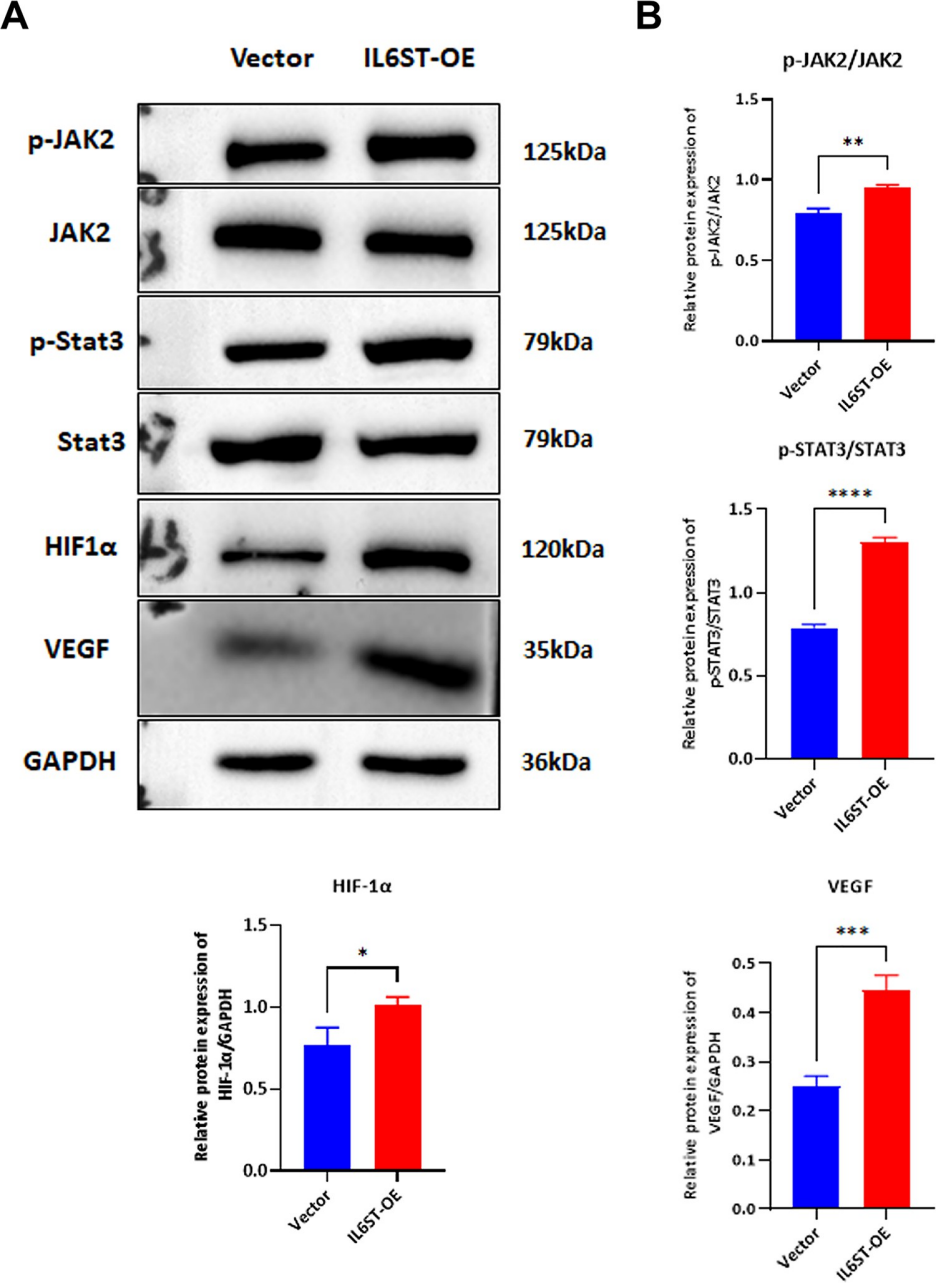
Regulation of the JAK2/STAT3 signaling pathway by *IL6ST* in endometriosis. (A) Western Blot analysis of pathway-related protein levels. (B) Image J analysis of band intensities. Data are presented as mean±SD. Statistical analysis was performed using t-tests, *P<0.05, **P<0.01, ***P<0.001.

To determine if *IL6ST* exerts regulatory influence on progression of endometriosis through JAK2/STAT3 signaling pathway, cells transfected with *IL6ST* overexpression vector exhibited strikingly elevated levels of p-JAK2/JAK2, p-STAT3/STAT3, HIF-1α, and VEGF proteins in contrast to cells transfected with the empty vector (Fig 3A and 3B). These data indicate that phosphorylation status of the JAK2/STAT3 signaling pathway is aberrantly activated, thereby fostering advancement of endometriosis.

### WP1066 inhibits the activation of the JAK2/STAT3 pathway by *IL6ST* overexpression

**Fig 4:**
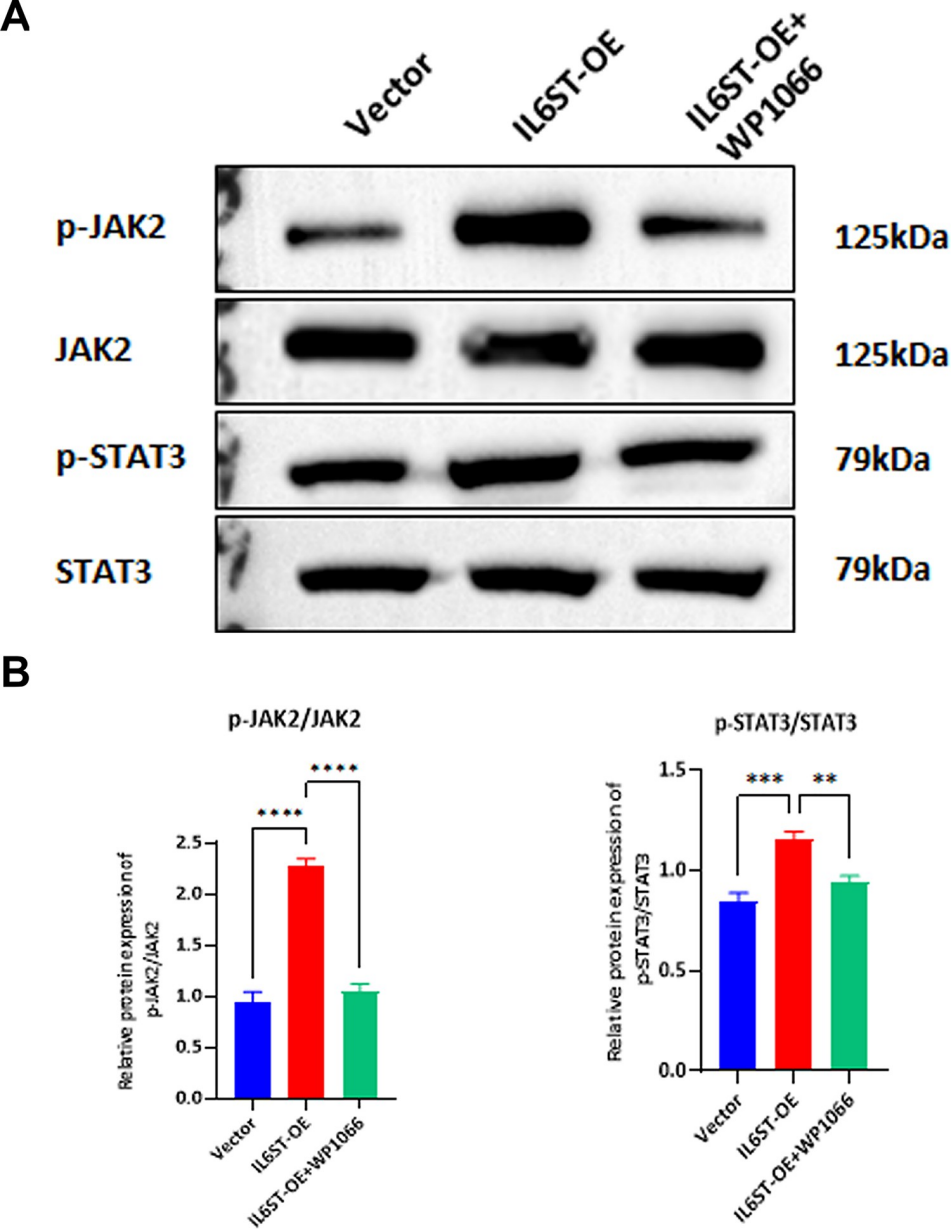
Inhibition of the JAK2/STAT3 signaling pathway by WP1066 in endometrial stromal cells. (A) Western Blot analysis of pathway-related protein levels. (B) Image J analysis of band intensities. Data are presented as mean±SD. Statistical analysis was performed using t-tests, **P<0.01, ***P<0.001, ****P<0.0001.

WP1066 is a novel, cell-permeable inhibitor of JAK2 and STAT3 phosphorylation, exerts no impact on JAK1 and JAK3. Culturing of *IL6ST*-OE endometrial stromal cells in presence of JAK2/STAT3 inhibitor WP1066 led to substantial reduction in the protein levels of p-JAK2 and p-STAT3 when compared to the *IL6ST*-OE group in isolation (Fig 4A and 4B). These findings support premise *IL6ST* overexpression facilitates progression of endometriosis by instigating activation of JAK2 and STAT3 phosphorylation.

### Inhibition of the JAK2/STAT3 pathway attenuates *IL6ST*-induced proliferation and invasion, while promoting apoptosis of endometrial stromal cells

**Fig 5:**
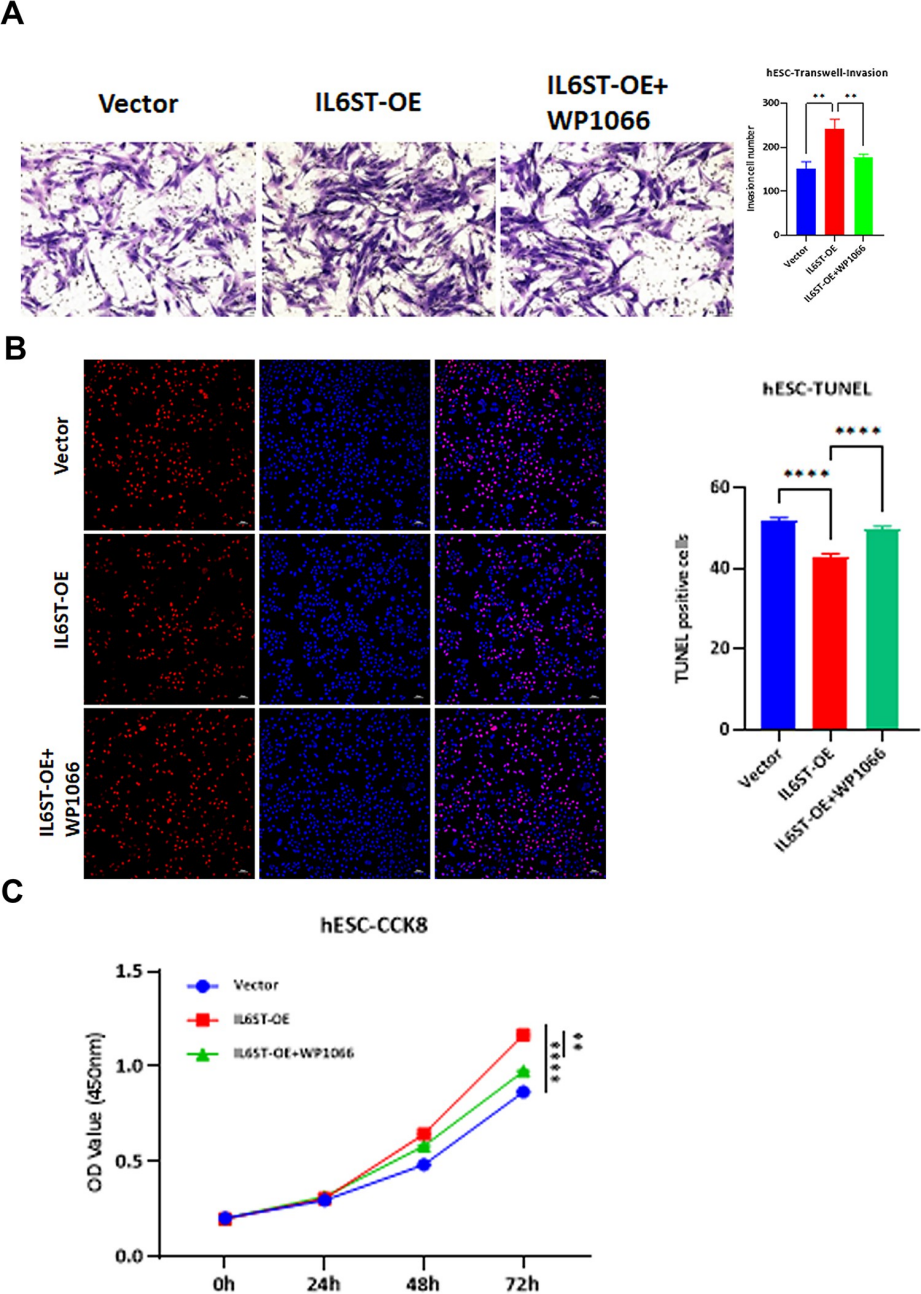
Effects of WP1066 inhibitor on the proliferation, invasion, and apoptosis of endometrial stromal cells. (A) Transwell assay of cell invasion. (B) TUNEL assay of cell apoptosis. (C) CCK8 assay of cell proliferation. Statistical analysis was performed using t-tests, *P<0.05, **P<0.01, ***P<0.001.

In light of *IL6ST*’s modulatory effect via the JAK2/STAT3 pathway, an examination of endometrial stromal cell behavior was undertaken. Transwell exhibited conspicuous reduction in number of invasive cells in the *IL6ST*-OE+WP1066 group relative to the *IL6ST*-OE group (Fig 5A). TUNEL assays indicated significant enhancement in number of apoptotic cells in *IL6ST*-OE+WP1066 group compared to *IL6ST*-OE group (Fig 5B). CCK8 assays showcased marked diminution in OD value at 450 nm within *IL6ST*-OE+WP1066 group juxtaposed to *IL6ST*-OE group (Fig 5C). These findings suggest inhibition of JAK2/STAT3 pathway could effectively mitigate proliferative and invasive effects of *IL6ST* on endometrial stromal cells, concurrently enhancing apoptosis.

## Discussion and conclusion

Endometriosis is chronic and widespread gynecological condition, classified into four types: peritoneal, ovarian, deeply infiltrating, and extensive. *IL6ST* is crucial cytokine signal transducer, playing pivotal role in regulating various physiological functions within body, including immune, hematopoietic, and nervous systems. Methodological Approaches including genome-wide methylation arrays, chromatin immunoprecipitation (ChIP-seq), genomic gene expression, and DNA methylation profiling were employed to identify and screen for aberrant methylated genes in endometriosis patients [23–25]. Methylation abnormalities of IL-12B promoter region and low-level methylation of pivotal genes, encompassing HOXA10, have been linked to the onset of ovarian endometriosis [26, 27]. Methylation status may be regulated by DNMT family of enzymes, which may play more significant role in development of endometriosis. Conversely, reduced methylation levels have been observed to correlate negatively with mRNA expression [28].

*IL6ST* plays pivotal role in pathogenesis of numerous diseases. In intrahepatic cholangiocarcinoma, *IL6ST* is associated with reduction in overall survival and an acceleration of cancer progression [29]. In chronic obstructive pulmonary disease, *IL6ST* plays role in signal transduction of JAK2 and IL-6. JAK activation facilitates phosphorylation and nuclear translocation of STAT3, subsequently enhancing the transcription of inflammatory factors. In cases of Takayasu arteritis and other related conditions, elevated level of *IL6ST* expression has been identified through single-cell sequencing of PBMCs extracted from patient tissues [30].

The present study employed immunohistochemistry to detect *IL6ST* expression in endometrial tissues from patients with ovarian endometriosis (EMs) and normal controls. Results indicated increased expression of *IL6ST* in endometria of EM patients in comparison to controls, with significant upregulation observed in ectopic endometrial tissues. This suggests that *IL6ST* plays critical role in development of endometriosis, potentially being activated at early stage of the disease and further upregulated during abnormal endometrial proliferation and malignancy. The reduction in *IL6ST* methylation levels among patients with endometriosis suggests notable elevation in *IL6ST* mRNA expression levels in these patients.

Interleukin-6 (IL-6) exerts its biological activity through two distinct molecules: IL-6R (interleukin-6 receptor) and gp130. Upon binding to mIL-6R (the membrane-bound form of IL-6R), IL-6 induces the dimerization of gp130, thereby forming a high-affinity functional receptor complex (IL-6/IL-6R/gp130). Additionally, soluble form of IL-6R (sIL-6R) can bind IL-6, and IL-6/sIL-6R complex similarly associates with gp130 to form complex. Homodimerization of these receptor complexes activates JAK (Janus kinase), which in turn leads to phosphorylation of tyrosine residues in gp130 cytoplasmic domain. Activation of gp130 by IL-6 gives rise to two principal signaling pathways: the SHP-2/ERK/MAPK pathway, which is derived from gp130 Tyr759, and the JAK/STAT pathway, which is mediated by gp130 YXXQ [31]. It has been demonstrated that diseases involving IL-6 and IL-6R are associated with etiology of endometriosis. An increase in sIL-6R levels in peritoneal fluid has been demonstrated to enhance biological activity of IL-6, thereby promoting development of endometriosis [32]. Presence of lactic acid has been observed to enhance progression of endometriosis by facilitating key processes such as cell invasion, angiogenesis, and immune suppression. Hypoxic conditions serve to exacerbate this process by upregulating HIF1A expression, while the elevated expression of VEGF serves to further accelerate angiogenesis [33]. In this study, the aforementioned cellular functional experiments collectively suggest that the stable overexpression of 3*FLAG-*IL6ST* augments the proliferation and invasion of endometrial stromal cells while mitigating their apoptosis to varying extents.

The present study demonstrates significant role of *IL6ST* in pathology of endometriosis through lentivirus construction, cellular behavior experiments, and protein-level detection experiments. Interleukin-6 (IL-6), an inflammatory cytokine, mediates signal transduction through its receptor, IL-6R, and co-receptor gp130 (*IL6ST*), thereby activating downstream Janus kinase (JAK)/signal transducer and activator of transcription 3 (STAT3) pathway. *IL6ST* has been demonstrated to enhance the proliferation and invasive capabilities of endometrial stromal cells, while simultaneously inhibiting apoptosis. This may be attributed to STAT3 binding to the promoters of HIF1A and VEGF, thereby promoting angiogenesis.

WP1066, STAT3 inhibitor, has demonstrated efficacy in mitigating broad range of inflammatory responses by inhibiting the JAK2/STAT3 pathway. Upregulation of long non-coding RNA (lncRNA) Stat3 has been shown to facilitate proliferation, migration, and invasion of endometrial stromal cells (ESCs) while simultaneously inhibiting apoptosis [34]. Macrophages, which rely on STAT3 activation, have been implicated in development of endometriosis [35]. Modulation of the JAK2/STAT3 signaling pathway has been demonstrated to enhance the migratory and invasive capacity of endometriotic cells by upregulating expression of matrix metalloproteinase-2 (MMP-2) [36]. Another area of interest is its promotion of mesothelial transformation in endometriosis [37]. In this study, WP1066 treatment of *IL6ST*-overexpressing cells demonstrated that WP1066 inhibited *IL6ST*-induced promotion of JAK2/STAT3 pathway. Inhibition of JAK2/STAT3 pathway resulted in reduction in the proliferation and invasion of endometrial stromal cells, accompanied by an increase in apoptosis.

IL-6ST/JAK/STAT signaling cascade plays cardinal role in progression of endometriosis, positioning it as promising therapeutic target. *IL6ST* serves as critical signal transducer for IL-6, and prior research has highlighted marked elevation in *IL6ST* expression in ectopic endometrial tissues compared to eutopic and normal tissues. These differential genes are predominantly concentrated in JAK-STAT signaling pathway. These differentially expressed genes are primarily clustered within JAK-STAT signaling pathway. These findings provide valuable insights into the pathogenesis of endometriosis. Knockdown of circ_0007331 has been shown to impede the progression of endometriosis by downregulating HIF-1α expression [38]. The selective silencing of vascular endothelial growth factor (VEGF) in vitro has been shown to inhibit cell proliferation, invasion, and migration [39]. Overexpression of VEGFC stimulates endothelial cell migration, increases vascular permeability, and promotes angiogenesis and EM development. In this study, *IL6ST*-overexpressing endometrial stromal cell lines were constructed, and functional changes that occurred as result of overexpression were examined. The results demonstrated that *IL6ST* overexpression markedly augmented cellular proliferation and invasion while concomitantly reducing apoptosis. Additionally, expression levels of proteins associated with the JAK2/STAT3 pathway, including p-JAK2, p-STAT3, HIF-1α, and VEGF, were found to be significantly elevated. This indicates that *IL6ST* stimulates the proliferation and survival of endometrial stromal cells at ectopic sites by activating the JAK2/STAT3 signaling pathway, thereby establishing *IL6ST* as potential therapeutic target for endometriosis. Previous studies have demonstrated that eutopic and ectopic endometrial stromal cells in endometriosis patients exhibit distinct invasive, adhesive, and proliferative behaviors, with *IL6ST*/JAK2/STAT3 pathway potentially exerting pivotal influence. Further research is required to elucidate the specific mechanisms of the IL-6/*IL6ST*/JAK2/STAT3 signaling pathway in endometriosis and to explore potential therapeutic methods targeting these genes.

## Supporting information

S1 Raw data (ZIP)

S1 Raw images (PDF)

## Abbreviation

IL6ST: Interleukin-6 signal transducer
gp130: Glycoprotein 130
JAK2: Janus kinase 2
STAT3: Signal transducer and activator of transcription 3
IHC: Immunohistochemistry
Ems: Endometriosis patients
CCK8: Cell Counting Kit-8
TUNEL: Terminal deoxynucleotidyl transferase dUTP nick end labeling
DAB: 3,3’-Diaminobenzidine
IL-6: Interleukin-6
sIL-6R: Soluble interleukin-6 receptor
NF-κB: Nuclear factor kappa B
PBMCs: Peripheral blood mononuclear cells
ERK: Extracellular signal-regulated kinase
MAPK: Mitogen-activated protein kinase
VEGF: Vascular endothelial growth factor
HIF1A: Hypoxia-inducible factor 1, alpha subunit
EC: Ectopic endometrial cells
