# Supplementary material for: Hypomethylation of IL6ST promotes development of endometriosis by activating JAK2/STAT3 signaling pathway
Source: PLoS One. 2025 Jan 16;20(1):e0317569. doi: 10.1371/journal.pone.0317569 (PMC11737718; doi:10.1371/journal.pone.0317569)

Fig2A.IL6ST overexpression verification-GAPDH

Raw blot (Left): Western blot analysis was performed on protein extracts derived from primary human endometrial stromal cells transfected with the VECTOR plasmid, utilizing gel imaging system software for capturing images.

Raw blot (Right): Western blot analysis was conducted on protein extracts obtained from primary human endometrial stromal cells transfected with the IL6ST plasmid, employing gel imaging system software for capturing images.

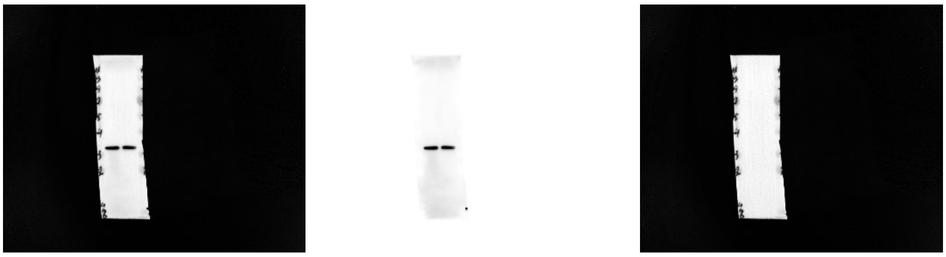

IL6ST overexpression verification-IL6ST

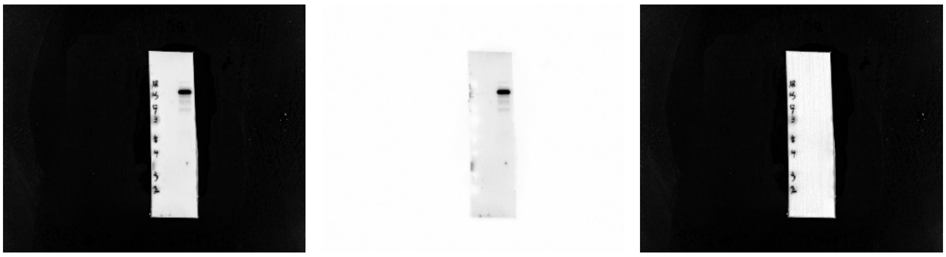

FIG2C.TRANSWELL detects the number of cells that are migrating

VECTOR

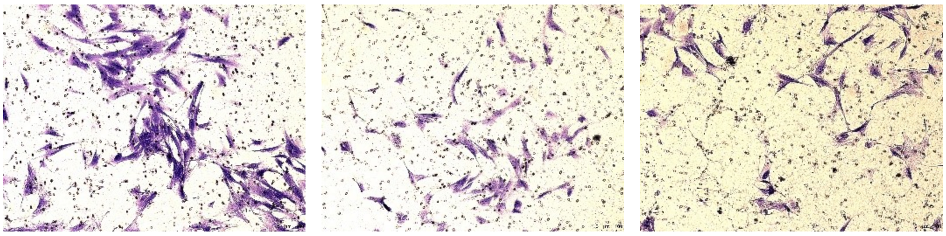

IL6ST-OE

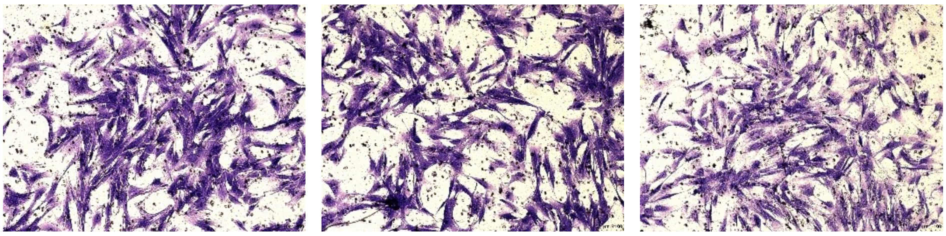

FIG2D.TUNEL for apoptotic detection

VECTOR

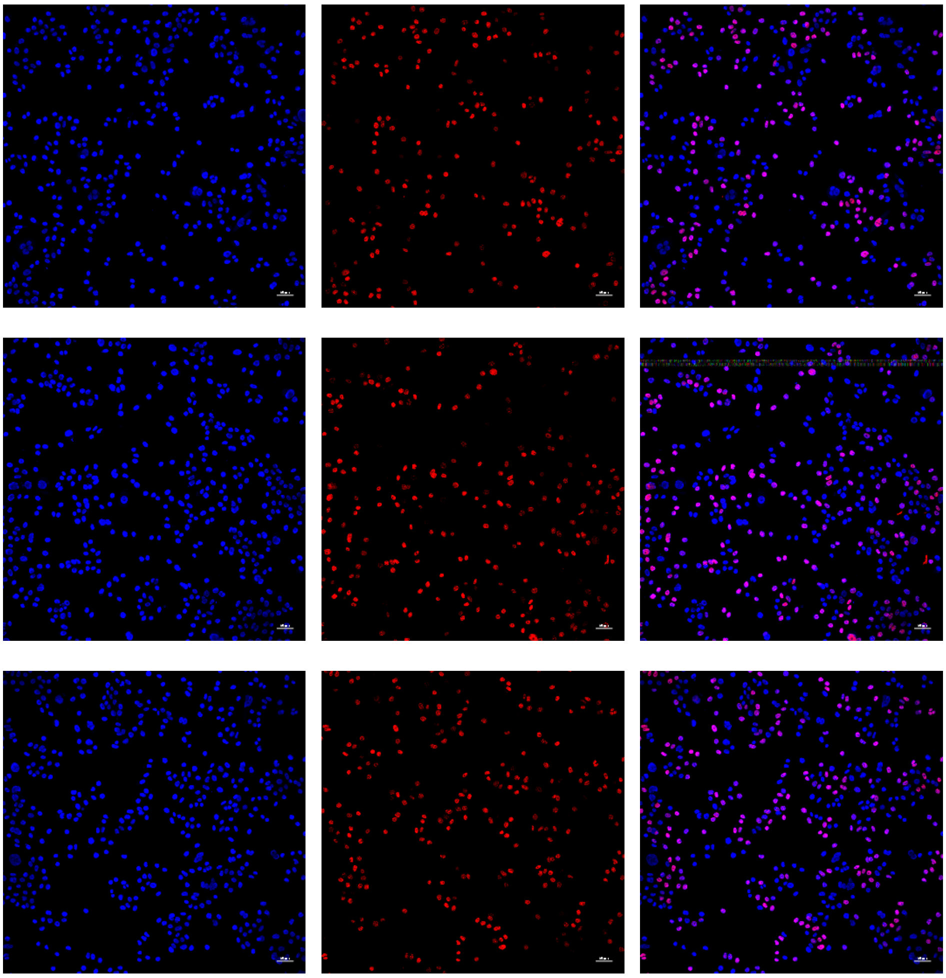

IL6ST-OE

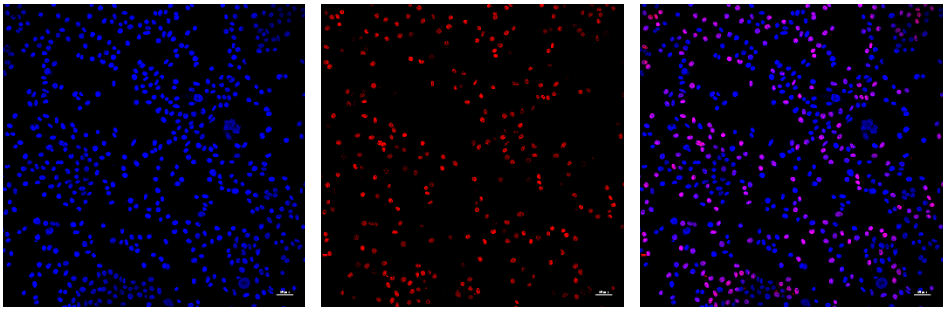

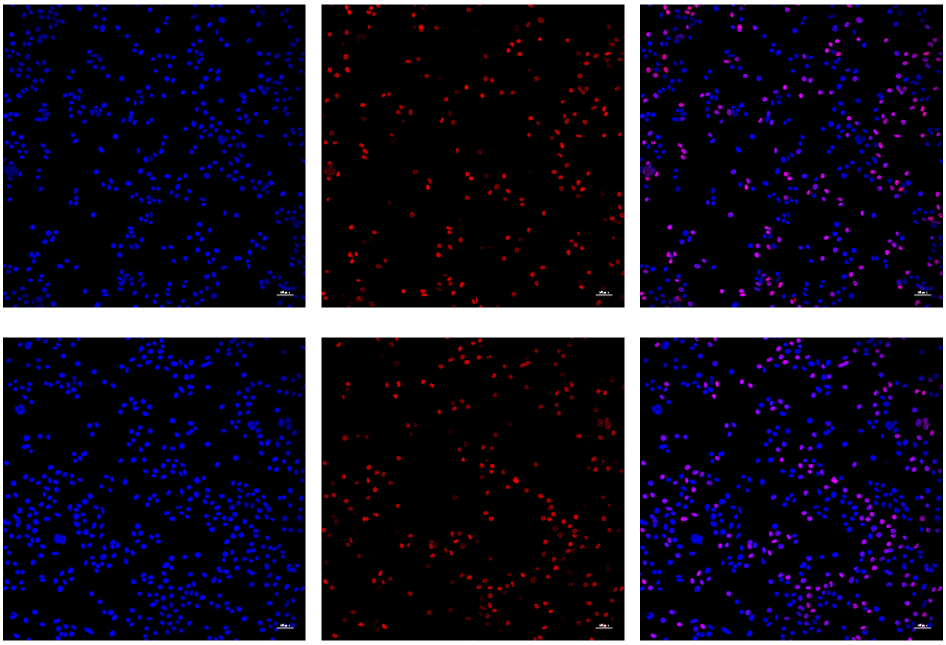

FIG3

VECTOR VS IL6ST-OE

Raw blot (Left): Western blot analysis was performed on protein extracts derived from primary human endometrial stromal cells transfected with the VECTOR plasmid, utilizing gel imaging system software for capturing images.

Raw blot (Right): Western blot analysis was conducted on protein extracts obtained from primary human endometrial stromal cells transfected with the IL6ST plasmid, employing gel imaging system software for capturing images.

VEGF

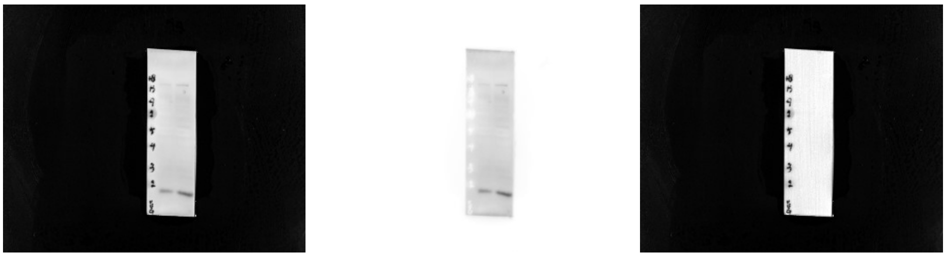

STAT3

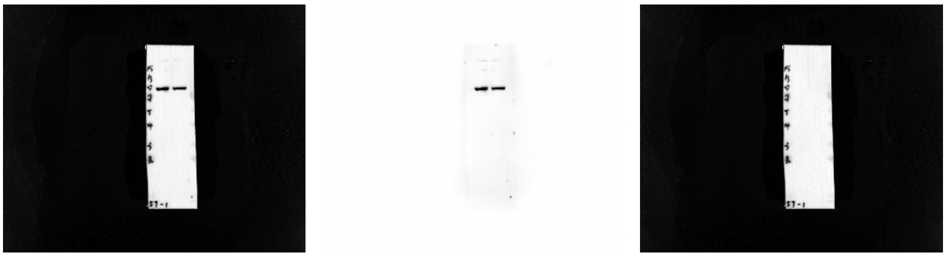

P-STAT3

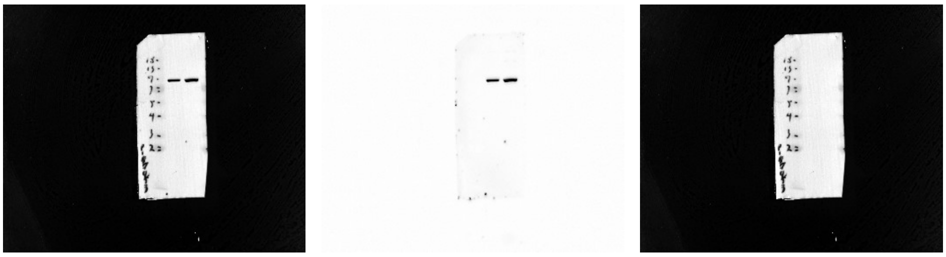

P-JAK

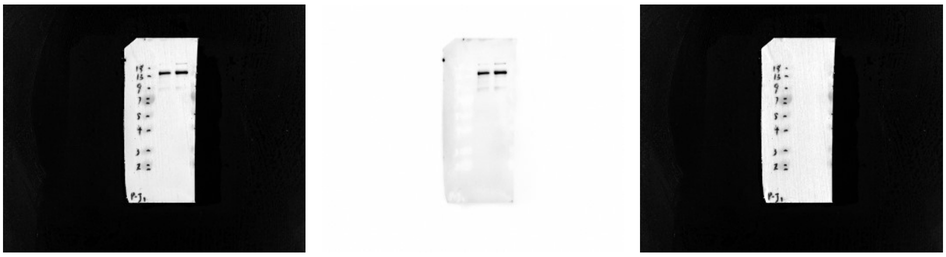

JAK

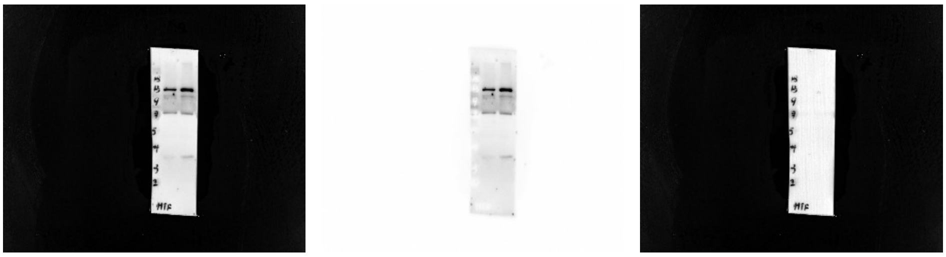

HIF1A

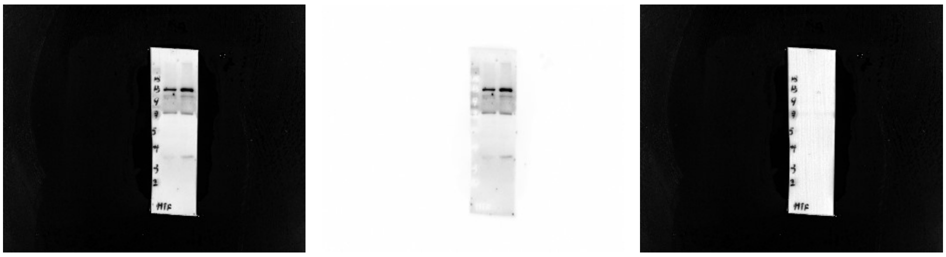

FIG4

Blank(vector)& IL6ST-OE& IL6ST-OE+WP1066

Raw blot (Left): Western blot analysis was performed on protein extracts derived from primary human endometrial stromal cells transfected with the VECTOR plasmid, utilizing gel imaging system software for capturing images.

Raw blot (Middle): Western blot analysis was conducted on protein extracts obtained from primary human endometrial stromal cells transfected with the IL6ST plasmid, employing gel imaging system software for capturing images.

Raw blot (Right): Western blot analysis was conducted on protein extracts obtained from primary human endometrial stromal cells transfected with the IL6ST plasmid and WP1066 added, employing gel imaging system software for capturing images.

JAK2

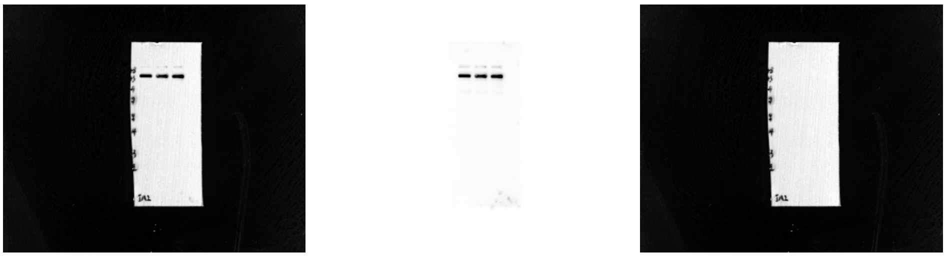

p-JAK2

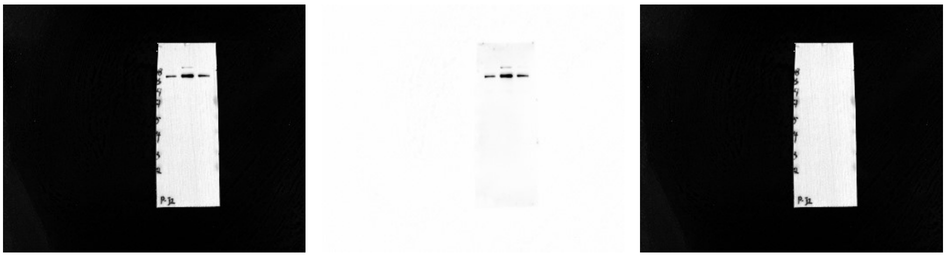

p-stat3

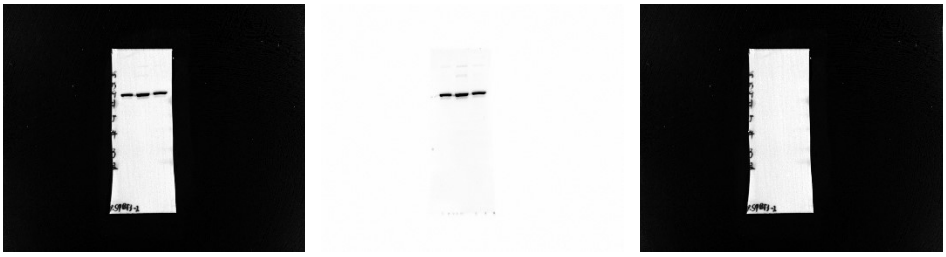

STAT3

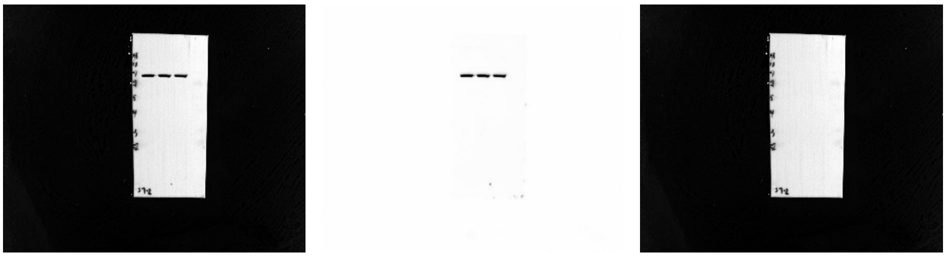

FIG5A

TRANSWELL: Blank(vector)& IL6ST-OE& IL6ST-OE+WP1066

Blank(vector)

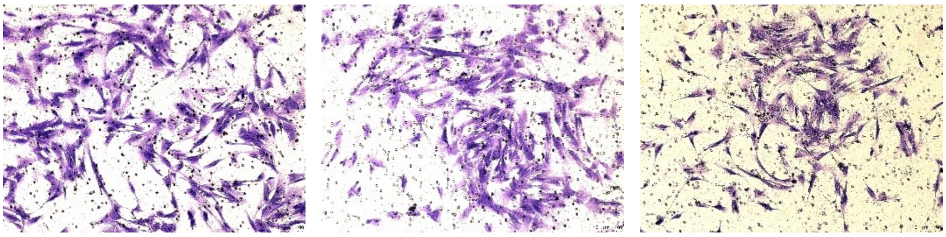

IL6ST-OE

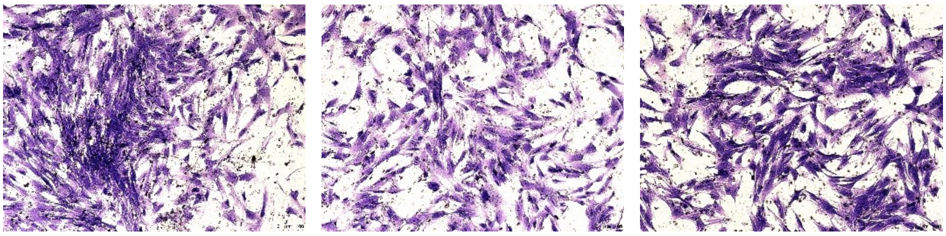

IL6ST-OE+WP1066

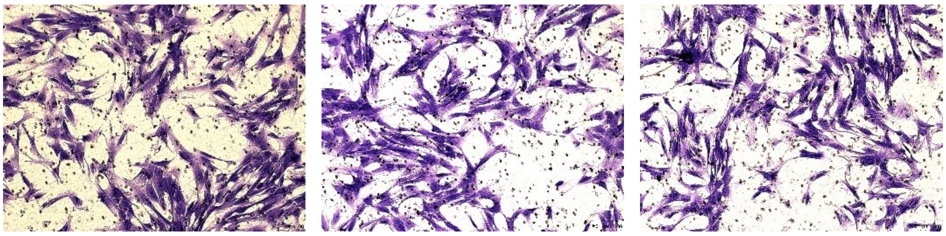

FIG5B

TUNEL: Blank(vector)& IL6ST-OE& IL6ST-OE+WP1066

Blank(vector)

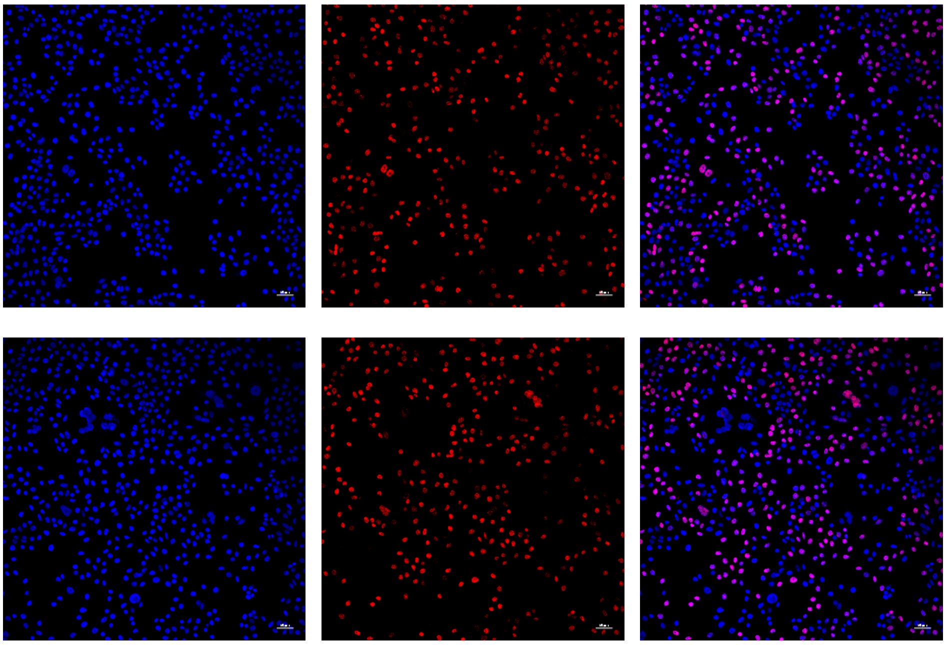

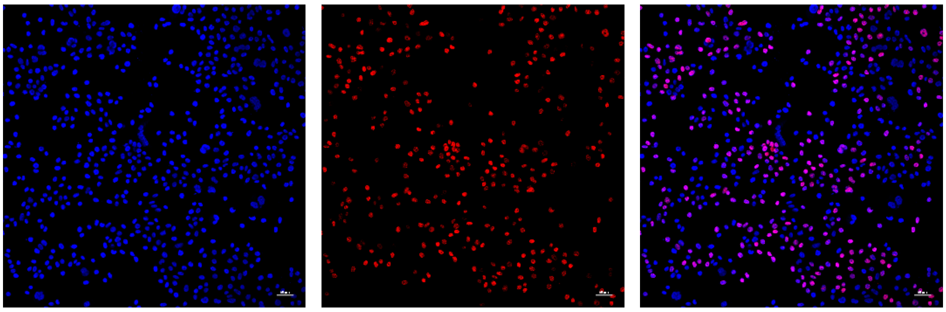

IL6ST-OE

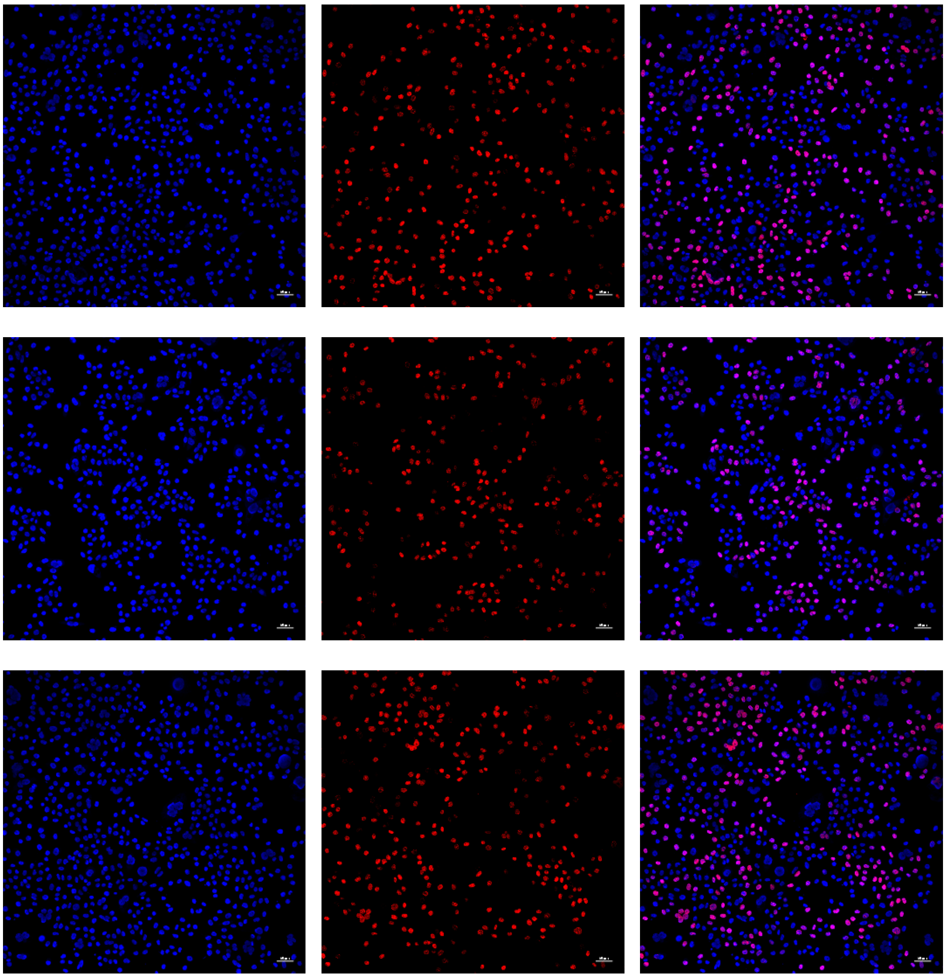

IL6ST-OE+WP1066

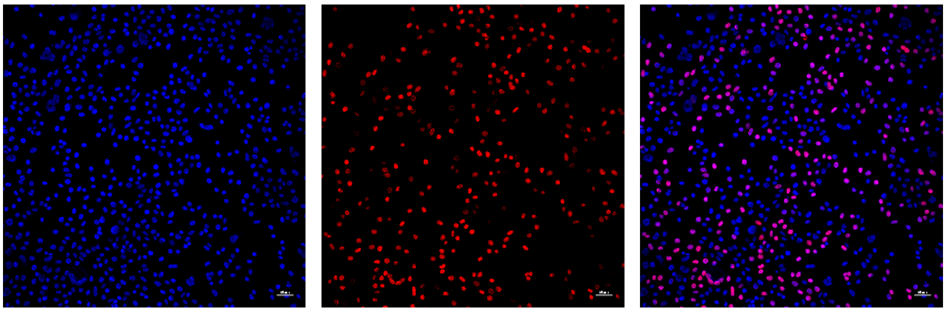

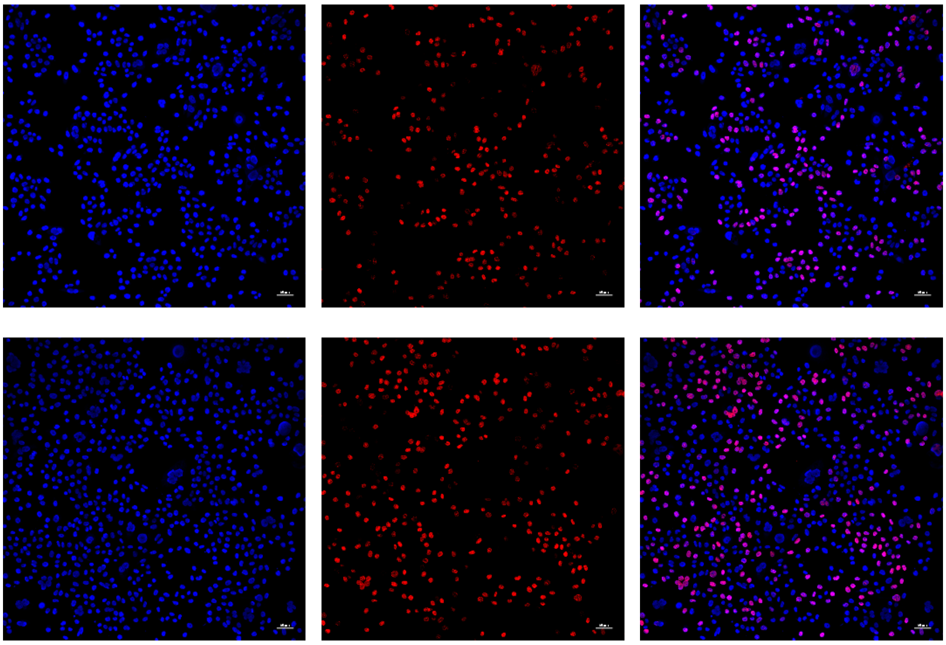

Supplement: S1 Raw images — (PDF) [file pone.0317569.s002.pdf]
